# Intracystic Primary Squamous Cell Carcinoma of the Breast: A Challenging Diagnosis

**DOI:** 10.1155/2016/6081634

**Published:** 2016-09-26

**Authors:** Vera Ramos, João Fraga, Teresa Simões, Margarida Figueiredo Dias

**Affiliations:** ^1^Gynaecology Service, Centro Hospitalar e Universitário de Coimbra, Coimbra, Portugal; ^2^Pathology Service, Centro Hospitalar e Universitário de Coimbra, Coimbra, Portugal

## Abstract

We report a case of a 36-year-old woman that presented with a painful mass in the outer quadrants of the left breast that had grown rapidly. Physical examination revealed a well circumscribed elastic mass and breast ultrasound showed a cyst measuring 26 mm with vegetation growing on the inner wall. Microscopic evaluation, after fine needle aspiration cytology (FNAC), suggested benign lesion. Tumorectomy was performed and the final diagnosis was a pure squamous cell carcinoma (SCC) of the breast. A simple mastectomy with sentinel node biopsy was performed. The histological study of the specimen revealed residual SCC and the sentinel lymph node was negative. The patient received 6 cycles of adjuvant chemotherapy and adjuvant radiotherapy. Four years later, the patient is free of disease.

## 1. Introduction

Metaplastic breast carcinoma (MBC) represents a rare and heterogeneous group of malignancies that accounts for less than 1% of all breast cancers [1]. Since the early reports MBC has been increasingly recognized and reported as a distinct histological type of breast cancer. The World Health Organization (WHO) classifies MBC into several types including squamous cell carcinoma (SCC) [2]. The lesions of the patients with MBC are often distinct from those of invasive carcinoma of no special type previously known as invasive ductal carcinoma (IDC) and are characterized by larger tumour size, less nodal involvement, higher tumour grade, and greater hormone receptor negativity [3]. SCC of the breast is an uncommon tumour and reported incidences are less than 0,1% of all breast carcinomas [4]. Clinical and radiologic features are not specific and in some cases SCC of the breast can even be mistaken for a benign disorder like a cyst or an abscess. Because of the rarity of these tumours it is difficult to establish what the best therapeutic approach is. We report a case of primary SCC of the breast presenting as an intracystic tumour. The case represents a challenging diagnostic investigation due to its presenting form and rarity.

## 2. Case Presentation

**Figure 1:**
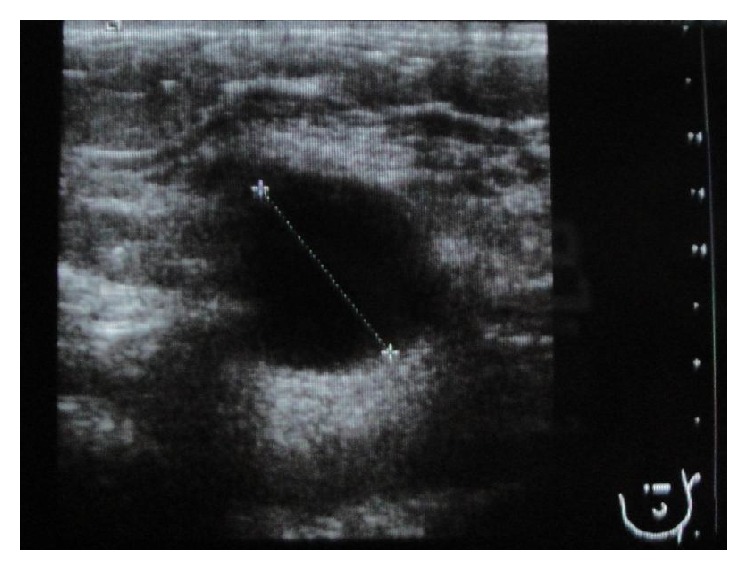
The cyst after fine needle aspiration cytology (FNAC).

**Figure 2:**
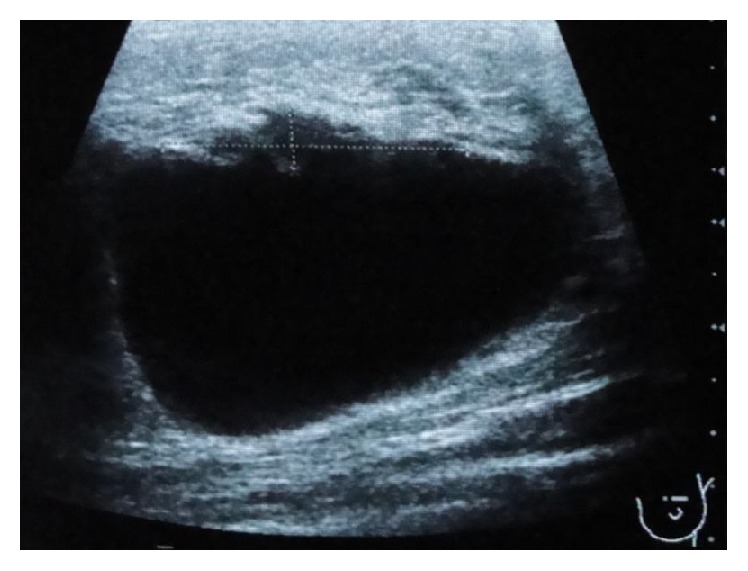
Ultrasound of the left breast (2nd evaluation): cyst measuring 44 mm with irregular and hypoechogenic vegetation of 6 × 27 mm.

**Figure 3:**
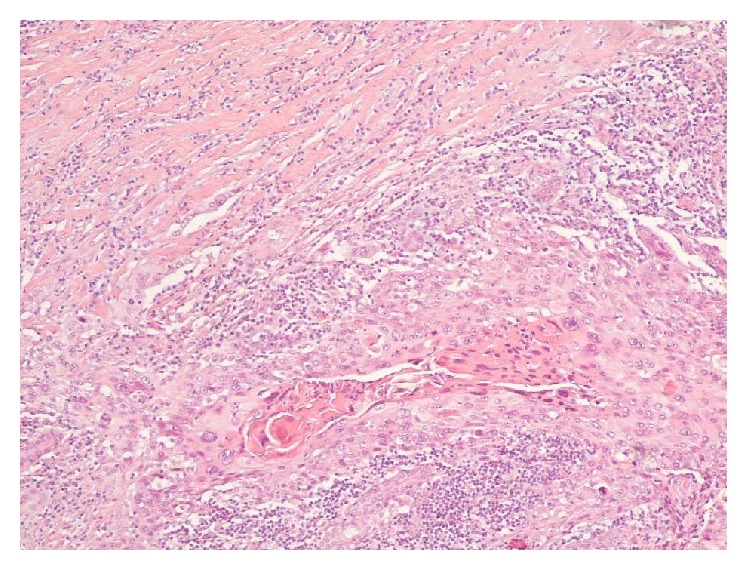
HE 100x nest and trabeculae of highly atypical epithelial cells, centrally with clear squamous differentiation.

**Figure 4:**
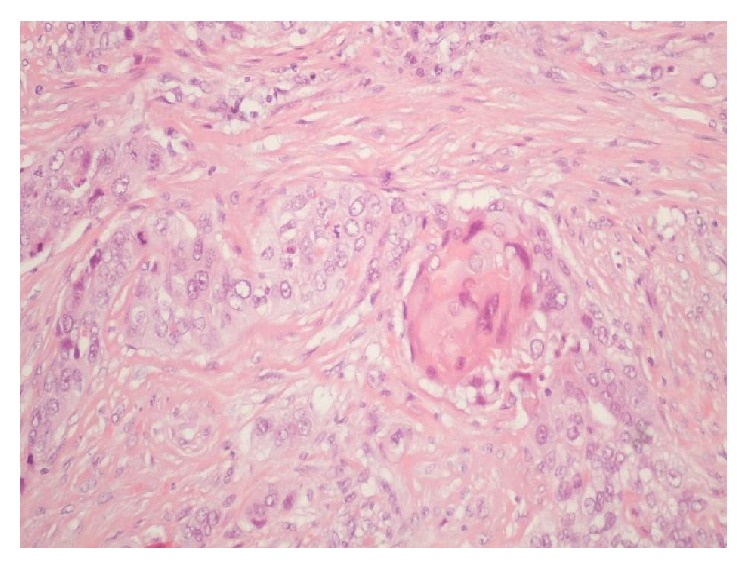
HE 200x invasive high grade carcinoma with squamous differentiation.

A 36-year-old premenopausal woman presented with a painful mass in the outer quadrants of the left breast that had grown rapidly for two months. The woman had no significant medical records and no family history of breast cancer. Her menstrual cycles were regular and she had three children. The initial physical examination revealed a well circumscribed elastic mass measuring about 30 mm not adherent to the underlying tissues or to the skin and no skin or nipple retraction was visible. Besides, the clinical examination did not reveal ipsilateral, axillary, or supraclavicular palpable lymph nodes. Nipple discharge was not evident. Contralateral breast and axilla were normal. The left breast ultrasound showed a cyst measuring 26 mm with irregular and hypoechogenic vegetation growing on the inner wall. A mammographic exam was not performed mostly due to the cystic appearance of the mass. Besides that there are no characteristic findings on mammography specific for this kind of tumours. The patient was submitted to fine needle aspiration cytology (FNAC) of the cyst and of the inner vegetation (Figure 1). The fluid obtained was yellowish and translucent and the size of the mass got reduced after the procedure. The smears were stained with Papanicolaou and May-Grünwald Giemsa stains. Microscopic evaluation revealed some foam cells and epithelial cells without atypia suggesting a benign cystic lesion. The biopsy was not performed since it was a cystic lesion, and, besides the inner vegetation, the whole ultrasonographic features and the FNAC results suggested a benign condition. One month later the breast ultrasound exam showed the persistence of the cyst, with increased volume, measuring about 44 mm maintaining the inner vegetation of 6 × 27 mm, which was irregular in shape and had continuity with the cyst wall (Figure 2). A surgery was performed because of the cyst rapid growth and ultrasound characteristics. At the time of hospitalization, one month after the second breast ultrasound, the physical examination revealed a painful large mass with 90 mm in the outer quadrants of the left breast. A breast tumorectomy was carried out. Macroscopically the surgical resection specimen, measuring 80 × 55 × 40 mm, showed a cyst. The inner surface of the cyst was mostly irregular with whitish vegetation. On histological examination a cystic lesion was seen, focally with atypical epithelial lining, with extensive ulceration. On the wall of the cyst there was a neoplastic infiltrative carcinoma with a solid pattern, high grade, with areas of squamous differentiation. Therefore, pure squamous cell carcinoma of the breast, grade 3, was diagnosed (Figures 3 and 4). It was difficult to establish the real size of the carcinoma because there was an intralesional section. The R classification was not given by the pathologist. The immunostaining for cytokeratin 34 beta E12 and p63 antibody was positive. Vimentin antibody and estrogen receptors were only positive in less than 5% of the cells. There was no reactivity for progesterone receptors or for HER2. The bone scan and thoracic-abdominal-pelvic computed tomography were negative as far as metastatic disease was concerned. The patient underwent surgery for a simple mastectomy with sentinel node biopsy. The decision was made taking into account the size of the tumour, the uncertainties about the residual tumour, and the preference of the patient. The histological study of the specimen revealed residual SCC reaching the deep margin with sentinel lymph node negativity regarding metastasis. The patient received 6 cycles of adjuvant chemotherapy (3 cycles of docetaxel and carboplatin followed by 3 cycles of fluorouracil, epirubicin, and cyclophosphamide). Adjuvant radiotherapy was performed to the chest wall in a standard regimen. Follow-up time is now four years and there is no evidence of disease. Cancer antigen 15.3 is negative. Imagiologic features of the left chest wall and right breast and axilla are normal.

## 3. Discussion

The pathological classification of pure SCC is challenging due to the diversity of histological pattern and the rarity of the diagnosis. It has been listed under metaplastic breast carcinomas according to the World Health Organization Classification [2]. The incidence of SCC of the breast reported in western countries is 0.1–3.6% and the age group more affected is between 32 and 65 years. Patients with MBC usually have larger primary tumours, higher histological grade, lower incidence of axillary node involvement, and lower incidence of hormone receptors positivity than patients with infiltrating ductal carcinoma (IDC) [3, 5, 6]; these characteristics are found in SCC reports as well [7, 8]. There are several theories about the origin of SCC of the breast; the most endorsed are related to epidermoid cyst of the breast, chronic abscess, and complete metaplasia of glandular breast tissue [9]. Squamous cells in FNAC of breast lesions can be found in various benign lesions, like epidermoid cyst, subareolar abscess, fibroadenoma, intraductal papilloma, spindle cell metaplasia, cystic sarcoma phyllodes, pseudosarcoma and malignant breast tumours, or metastatic malignancy [10]. Benign breast conditions containing abundant squamous cells may sometimes mimic malignant squamous lesion and vice versa. In general, the presence of abundant foamy macrophages in the background suggests a benign lesion. Benign squamous cells are bland looking and are often associated with anucleated squames. Malignant squamous cells are more pleomorphic, mitotically active, and dyskeratotic and, sometimes, bizarre-shaped cells can be seen [11]. The differential diagnosis of malignant squamous cells in FNAC of the breast includes primary SCC and metastasis. Careful assessment of cytological features of squamous cells and the background appearance is essential for achieving a truthful diagnosis. Some patches of squamous cells can be found in adenocarcinoma of the breast and from metastasis of squamous cell carcinoma that originated elsewhere. Metastasis spreading to breast commonly occurs from malignant melanoma, lymphoma, lung and ovarian carcinomas, soft tissue sarcoma, and gastrointestinal and genitor-urinary malignancies in order of decreasing frequencies. Metastases to the breast need to be considered if the histological appearance is unusual for a primary mammary tumour. Two-thirds of metastases to the breast have histological features, raising the possibility of this diagnosis. In some cases the histological appearance is similar to a primary mammary tumour and the clinical history is essential for making the diagnosis [12–16]. However, on what concerns intracystic SCC, numerous foamy macrophages coexist with malignant squamous cells. No characteristic findings on mammography are specific for this tumour explaining the advanced disease stage usually seen at diagnosis. Breast ultrasound has been reported to be more helpful as these tumours present as solid hypoechogenic masses with complex cystic components [17]. Because of its rarity the most appropriate therapeutic regimen for SCC of the breast is still unclear. The clinical behaviour of this tumour is uncertain. Breast SCC is usually a high grade and hormone receptor-negative tumour. This means that hormone-based therapy may not be effective [18, 19]. The lack of hormone therapy as a therapeutic option for adjuvant treatment combined with an increased risk of systemic metastasis that would be predicted given the large tumour characteristics explains the increased aggressiveness of treatment [3]. Nevertheless, data supporting the effectiveness of systemic chemotherapy for these patients are lacking and it is difficult to draw conclusions about the impact of this approach in the absence of randomized trials. SCC of the breast is reported to be resistant to standard chemotherapy performed for IDC such as methotrexate, cyclophosphamide, 5-fluorouracil (5-FU), and anthracycline [17, 20]. The role of radiation has been reported as unclear in many studies; although SCC are generally radiosensitive, locoregional relapse occurred frequently also in irradiated field. It seems that SCC of the breast is often relatively radioresistant [8]. Most patients with MBC and so patients with SCC, in reported series, have been treated with some form of mastectomy mainly because of the tumour size [3]. However, Teerthanath et al. suggested that the patients treated with breast conservative surgery experience similar local control and survival outcomes to those treated with mastectomy [21]. The surgical approach in SCC should also take into account the low rate of axillary involvement at presentation so the sentinel node biopsy seems to be more appropriate than routine axillary dissection [8]. Dave et al. postulated that the presence of skin invasion, at age not exceeding 39 years, and the presence of a squamous cell carcinoma component in the lymph nodes seemed to be the most important outcome predictors for patients with MBC [5]. The calculated overall 5-year survival rate for SCC varies from 63% to 67% according to retrospective different small series [7, 18]. The present case illustrates the main features of a SCC as reported in other small series. However, the intracystic form at presentation and the results of FNAC smears were in favour of a benign lesion. Careful assessment must be made considering the breast tumours, even cystic ones, particularly those of rapid growth. Clinical and pathological characteristics of SCC remain to be fully defined, so optimal treatment and prognosis are still unclear.
